# TACITuS: transcriptomic data collector, integrator, and selector on big data platform

**DOI:** 10.1186/s12859-019-2912-4

**Published:** 2019-11-22

**Authors:** Salvatore Alaimo, Antonio Di Maria, Dennis Shasha, Alfredo Ferro, Alfredo Pulvirenti

**Affiliations:** 1 Department of Clinical and Experimental Medicine, University of Catania, c/o Dipartimento di Matematica e Informatica, Viale A. Doria 6, Catania, 95125 Italy; 2 Department of Physics and Astronomy, University of Catania, Viale A. Doria 6, Catania, 95125 Italy; 3 Courant Institute of Mathematical Science, New York University, 251 Mercer St, New York, 10012 USA

**Keywords:** RNA-Seq, Cloud storage and management, Galaxy

## Abstract

**Background:**

Several large public repositories of microarray datasets and RNA-seq data are available. Two prominent examples include ArrayExpress and NCBI GEO. Unfortunately, there is no easy way to import and manipulate data from such resources, because the data is stored in large files, requiring large bandwidth to download and special purpose data manipulation tools to extract subsets relevant for the specific analysis.

**Results:**

*TACITuS* is a web-based system that supports rapid query access to high-throughput microarray and NGS repositories. The system is equipped with modules capable of managing large files, storing them in a cloud environment and extracting subsets of data in an easy and efficient way. The system also supports the ability to import data into Galaxy for further analysis.

**Conclusions:**

*TACITuS* automates most of the pre-processing needed to analyze high-throughput microarray and NGS data from large publicly-available repositories. The system implements several modules to manage large files in an easy and efficient way. Furthermore, it is capable deal with Galaxy environment allowing users to analyze data through a user-friendly interface.

## Background

Transcriptome analysis can be applied to define biomarkers in precision medicine, to describe how observed alterations impact a patient’s phenotype, or, more fundamentally, to infer causality among genes. To date, studies have produced a huge amount of data stored in databases such as NCBI GEO or ArrayExpress.

ArrayExpress [1] is a public database storing high-throughput functional genomics data, such as Microarray and Next-Generation Sequencing (NGS). Each dataset is provided by a user, either by submitting it directly, or by importing it from other databases (i.e. Gene Expression Omnibus, GEO). Directly submitted datasets are manually curated according to the MIAME (Minimum information about a Microarray experiment) standard [2] for Microarray, and MINSEQE (Minimum Information about a high-throughput nucleotide SEQuencing Experiment) standard for NGS.

These information standards support the sharing and reuse of scientific data. The MIAME standard was introduced in 2001 to simplify storing and exchanging gene expression experiments. The specifications of this standard requires the recording of all the information needed to unambiguously interpret the results of an experiment, and be able to reproduce the experiment. The standard defines the content and structure of necessary information, rather than the technical format for archiving. Similarly, MINSEQE defines a standard to allow the unambiguous interpretation and reproducibility of sequencing experiments.

Users who submit data to ArrayExpress provide their files together with metadata, describing sample content. Data are exported and stored according to the MAGE-TAB format [3]. Data sets imported from other functional genomics databases are also converted into MAGE-TAB files for archiving.

MAGE-TAB is a tabular MIAME file format. MAGE-TAB documents consist of five different types of files: (i) A ’raw’ archive; (ii) A ’data matrix’ file; (iii) The Sample and Data Relationship Format (SDRF) tab-delimited file; (iv) The Array Design Format (ADF) tab-delimited file; (v) The Investigation Description Format (IDF) tab-delimited file.

Retrieving publicly available data from ArrayExpress for analysis is a time-consuming task. First, some datasets are quite large so take time to download. Second, few tools are available to interact with ArrayExpress, though some are very good: in [4] authors presented the ArrayExpress package for R/Bioconductor to query ArrayExpress and convert MAGE-TAB formatted datasets from the ArrayExpress repository into objects of the Bioconductor class (eSet).

NCBI GEO is a public functional genomics data repository [5, 6] supporting MIAME-compliant data extraction. The system’s tools support user query and download experiments and curated gene expression profiles. End users may encounter problems when downloading large datasets.

To our knowledge, currently no system allows users to select small amounts of data from big experimental files. Consequently, users spend a lot of time to prepare data for further analysis. In addition, uploading data into Galaxy is complicated by the limited set of plugins offered by the platform and the need to manually pre-process external data.

Our system *TACITuS* (Transcriptomic Data Collector, Integrator, and Selector) is a web app that simplifies the process of collection, pre-processing, selection, and integration of transcriptomics data. Through our interface a user can collect data from major sources, such as NCBI GEO or ArrayExpress, and integrate them with their own data into a standardized format, facilitating subsequent analyses. Our tool, built on top of MongoDB, Apache Lucene, and proper indexing algorithms, can easily manage large amounts of data guaranteeing fast performance. In its current version, *TACITuS* supports only pre-processed NGS and Microarray dataset. Raw datasets can not be imported. Furthermore, all metadata are collected and standardized, enabling fast search and easy management on large datasets. Our software is freely available at https://tacitus.app/ and distributed through a GPL v3 licence. Finally, a module enabling complex data analysis through a connection with the Galaxy [7] computational platform is available.

To evaluate our methods, we imported several high dimensional transcriptomics datasets to determine efficiency in storage, pre-processing, and indexing. On such datasets, we also performed several selection queries and gathered their results to demonstrate the user experience. These experiments show that the user can import large datasets in a few hours, select samples in less than an hour, map and integrate datasets in a few minutes. All this is done with low memory and CPU consumption, making the software capable to scale and therefore suitable when large number of users are connected.

## Implementation

*TACITuS* is built on top of the Laravel framework and is backed by two databases: MariaDB for storing fast indexes of all available datasets, and MongoDB for data and meta-data storing. Data processing is done in R, C++ and PHP to achieve high performances. Our portal collects data from the major data sources, NCBI GEO and ArrayExpress, and integrates them with user data. *TACITuS* offers five major functionalities: (i) data import, (ii) data selection, (iii) identifier mapping, (iv) data integration, and (v) Galaxy Export. In this section, we will provide a description of these modules and their implementation. For more details, an online step-by-step tutorial is available through the web interface.

### Import

**Fig. 1:**
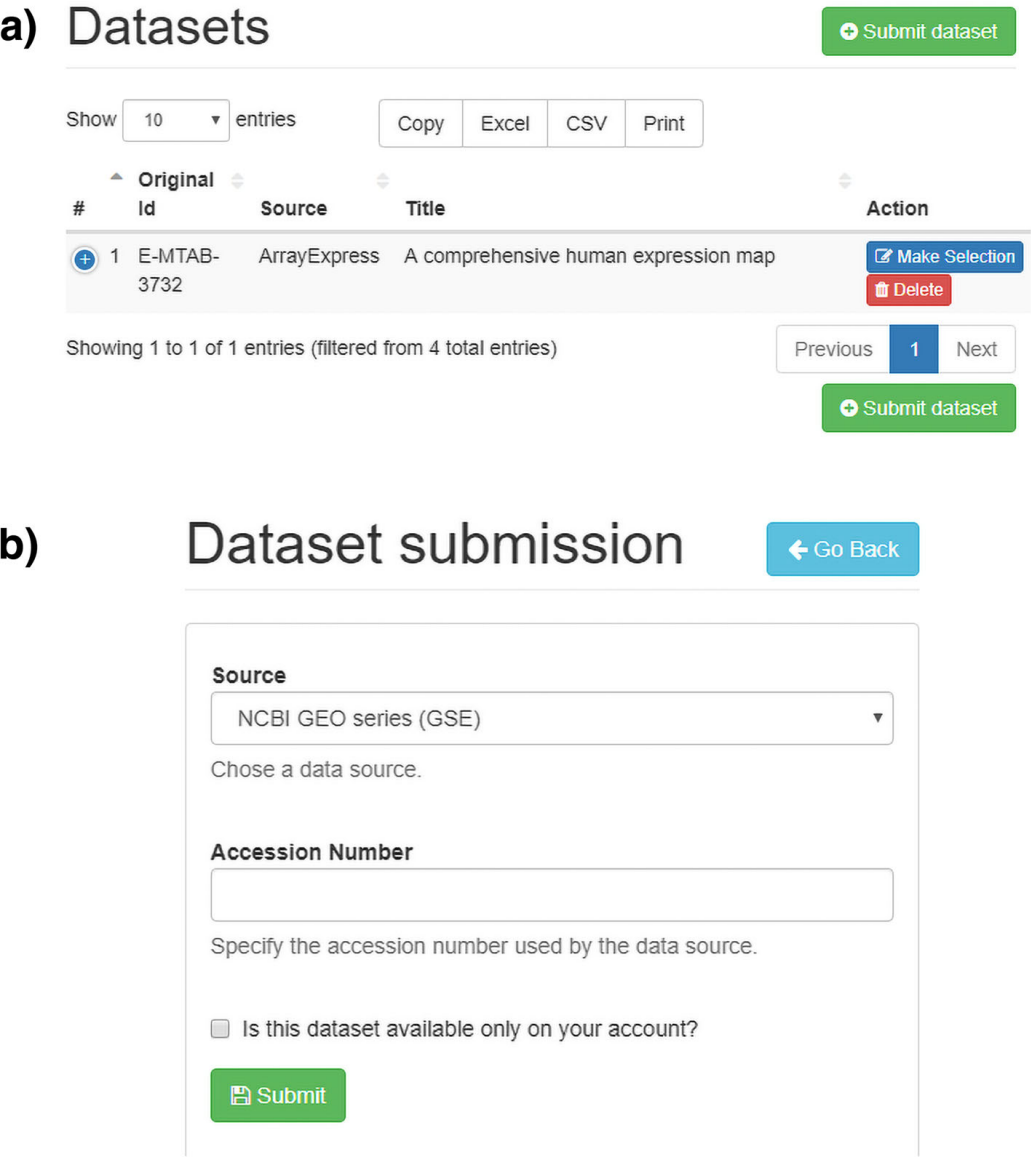
Data import panel. **a** The user selects the “Submit Dataset” button in the “Datasets” panel. **b** By filling the “Dataset submission” form an import request can be submitted

*TACITuS* enables users to import dataset from several public transcriptomics resources. Through the “Dataset submission” panel (see Fig. 1a), a user selects one data source (NCBI GEO, ArrayExpress or custom), specifies the accession number of the dataset to be imported (see Fig. 1b), and decides whether imported datasets should be public or private. In the current interface, NGS and Microarray datasets are supported if processed data are available. Raw datasets can not be imported in this release.

When the request is submitted, a record is added into a priority queue with all user-specified parameters and the computation starts as soon as resources are available.

First, dataset files are downloaded. Next, all relevant data are stored in a MongoDB databases, while building two indexes to speed-up further phases: (i) a meta-data index, and (ii) a sample index. The meta-data index is a list of all sample descriptor attributes. The sample index is a map that associates samples to their positions in the expression matrix. Samples are identified by a unique code. Finally, for each meta-data attribute, a Lucene-backed full-text index is built and a record describing the dataset is stored in the database and made available to the user.

For NCBI GEO datasets, the platform descriptor, containing information on mappings between probes identifiers and the respective ones in other datasets, such as Entrez or Ensembl Gene Ids, is also downloaded and imported in the database.

### Selection

**Fig. 2:**
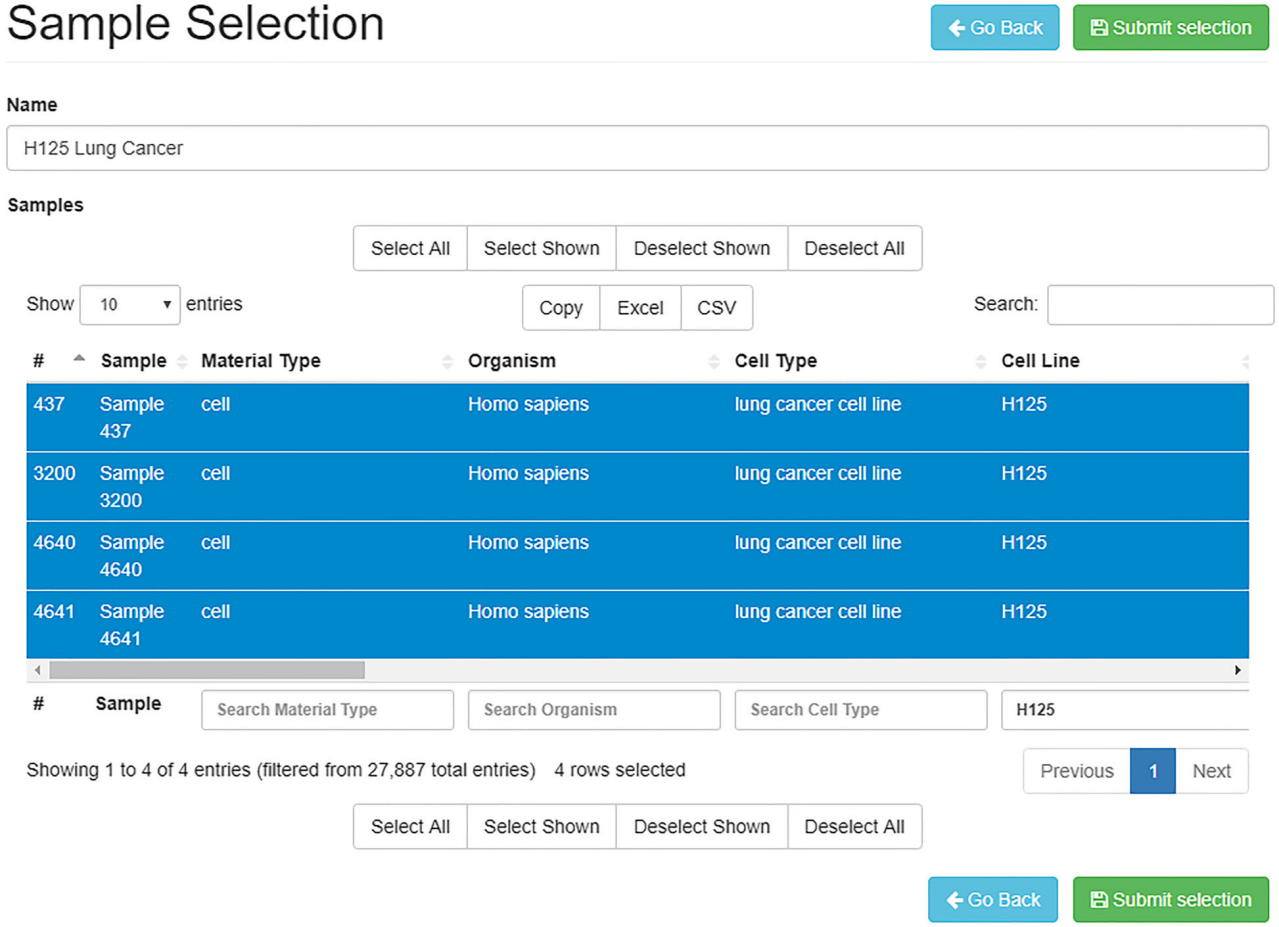
Data selection panel. The “Sample selection” panel can be employed to produce a sub-datasets containing only portion of the samples. The selection table provides functions to filter samples by metadata attributes

*TACITuS* allows users to select portions of imported datasets through a meta-data search. Selection improves performance when working with large datasets that can not be entirely loaded into memory. By clicking on the “Make Selection” button in the list of datasets (see Fig. 1a), the “Sample Selection” panel will appear, allowing to the user to specify all parameters. Specifically, the user can provide a selection name, for easy content identification, and select the samples to include. The table lists all samples and their meta-data to simplify the selection process. Sample search by meta-data is supported by the full-text indices built during the import phase (see Fig. 2).

After submitting the selection, a job is added to a priority queue, and processed when computational resources become available. As the process starts, selected sample meta-data are stored in a TSV (Tab-Separated Value) file and positions of such samples in the dataset expression matrix are extracted from the sample index. Next, all probes containing each sample set are gathered and stored in TSV files. Each line of the file contains the name of a probe, and the selected expression values. The first line of such a file contains the name of the selected samples.

As soon as the selection process is completed, the user can download both data and metadata in TSV or CSV format. If CSV format is chosen, the delimiter character can be specified.

### Mapping

For each selection, *TACITuS* supports a mapping from probe identifiers to standardized, e.g., Entrez, Identifiers. This facilitates the data integration process when using different platforms, because standardized identifiers between different experiments can be easily matched.

**Fig. 3:**
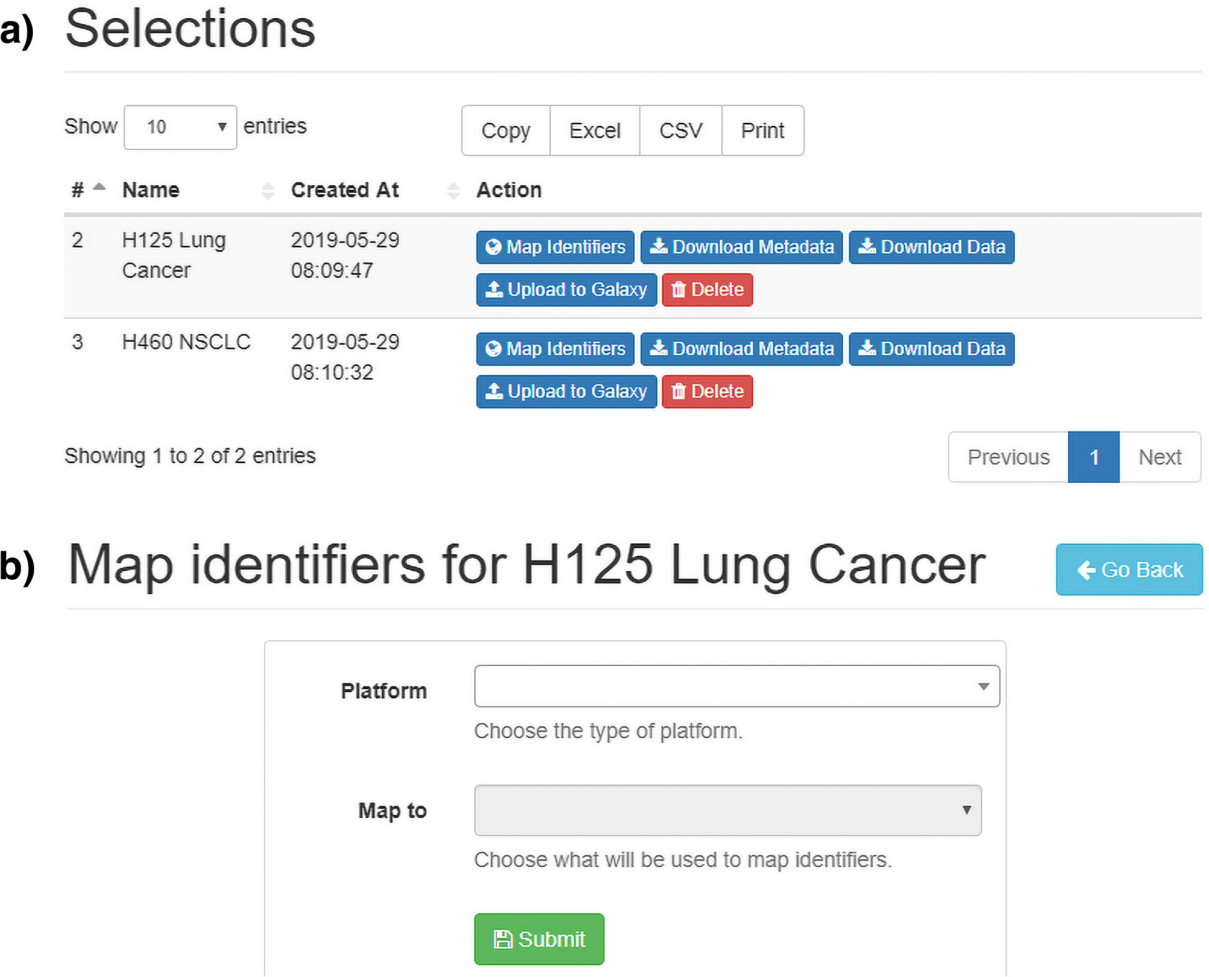
Identifiers mapping panel. **a** The user selects the “Map Identifiers” button in the “Selections” panel. **b** By choosing a platform and a destination identifier in the “Map Identifiers” form a request is submitted

The functionality is activated through the “Selections” panel, where the “Map Identifiers” button is located (see Fig. 3a). By making such a selection, a panel will open up. The desired platform for the transcriptomics experiment and the destination identifier are then selected (see Fig. 3b). If, as in NCBI GEO, the platform is automatically detected, the user can proceed directly to the destination identifier choice.

Once the request has been completed, a job is added to the priority queue. As soon as processing starts, a mapping table is built, where each probe identifier is associated with a standard one. The data file is read, and each probe identifier is replaced with the selected one, according to the mapping table. If no entry is available for an identifier, the probe will be removed from the output. The results are stored in TSV format. The first line contains the selected sample names, while the remaining lines contain the expression of probes for which a mapping is available. The user can choose to download data and metadata in TSV or CSV format.

### Integration

*TACITuS* implements an integration procedure devised to combine two or more selections into a single dataset. The system exploits the following techniques: (i) Sims et al., 2008 [8], (ii) COMBAT [9], (iii) Gene Standardization, and (iv) Cross-platform Normalization (XPN) [10].

Sims et al., 2008 applies a technique like z-score normalization, transforming each dataset through mean-centering. COMBAT exploits an Empirical Bayesian model to estimate the mean and variance of each gene in each dataset, correcting data for batch effects. Gene Standardization is the simplest mathematical transformation to make datasets comparable. For each gene, the expression value is corrected subtracting the mean and dividing by the standard deviation. XPN finds blocks of genes and samples showing similar expression patterns and, therefore, uses the average of these blocks to shift and scale data.

**Fig. 4:**
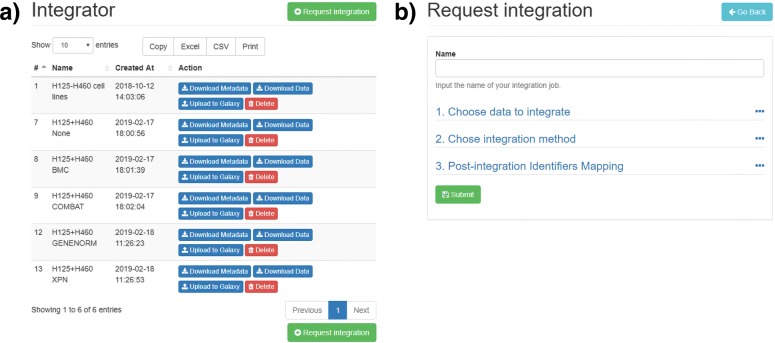
Integration panel. **a** The user requests the integrator procedure through the button in the “Integrator” panel. **b** The request can be submitted after choosing the dataset selection and the algorithm

The integration procedure starts by clicking on the “Request Integration” button available through the “Integrator” panel (see Fig. 4a). The user specifies which datasets to integrate, the combination method (see Fig. 4b). Integration algorithms can also be disabled if the user needs to combine expression matrices without altering the values.

Once the request has been completed, a job is added to the priority queue. The process will start as soon as the resources are available. First, all meta-data are merged in a single table, using the meta-data index to speed-up the identification of variables in the final table. All attributes from the original datasets are saved. If one or more samples in the merged table have the same identifier, a unique number will be appended. Next, all expression matrices are loaded in memory and the chosen algorithm builds a combined expression matrix. Finally, results are stored in TSV format and can be downloaded either in TSV or CSV format.

The integration procedure loads full expression matrices, therefore is a memory-intensive process.

### Galaxy integration

**Fig. 5:**
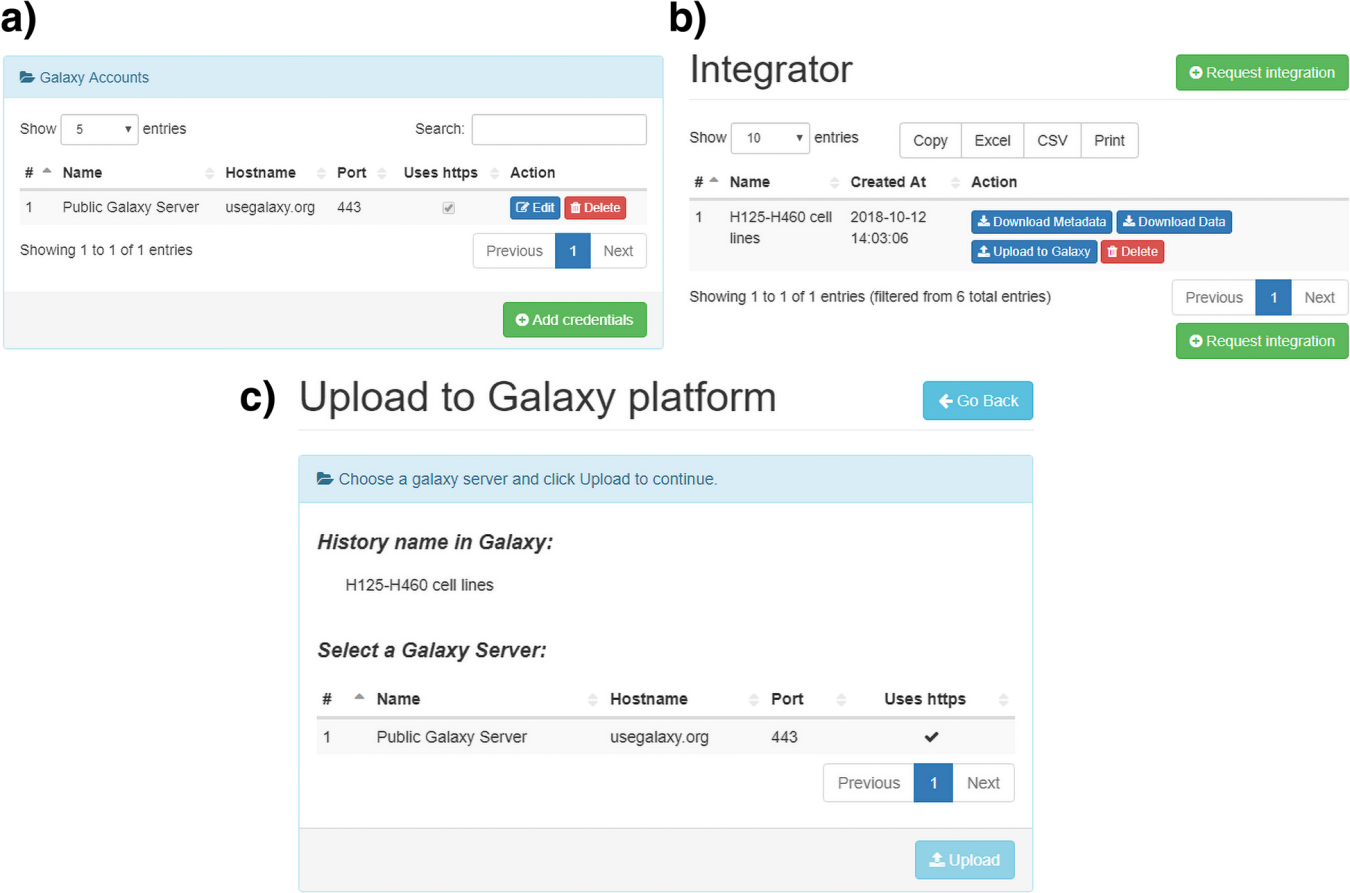
Galaxy Upload Module. **a** The module is activated by adding at least one server in the “Galaxy Accounts” panel. **b** The user will be then able to use the “Upload to galaxy” button provided in all panels. c) Finally, the upload process can be initiated in the “Upload to Galaxy” panel

*TACITuS* implements a module to upload data and metadata to the Galaxy platform. The integration with the Galaxy cloud environment has been done with Blend4php API [11]. To enable this module, the user has to provide the details of a galaxy server (name, host name, port) together with user’s credentials (API key) through the “Galaxy Account” panel in the “User Profile” page (see Fig. 5a). This activates the “Upload to Galaxy” button (see Fig. 5b). By clicking on the upload button beside each dataset, the user will open the uploading panel, enabling the selection of a Galaxy server for the upload (see Fig. 5c).

Once the selection has been made, the request is submitted to the priority queue and the process will start as soon as the requested resources become available. First, a connection to the remote Galaxy Server is created. Then an authentication request through the user API key is performed. If the request is successful, a new *history* with the dataset name is created. An history is a container in Galaxy maintaining the location of a dataset and the results of its analysis. Finally, data and metadata files are sent to the galaxy server for storage in the generated container.

## Results

To show how our approach can manage large expression data, we perform a case study with the ArrayExpress dataset “A comprehensive human expression map” (E-MTAB-3732). This dataset has been selected because of its size and complexity (~ 30*GB*). The dataset consists of 27,887 samples comprising expression data from approximately 50,000 genes. All experiments were performed using the Affymetrix HG-U133Plus2 platform. For each sample descriptive variables identifying cell type, source tissue, cell line, and any pharmacological treatments are available. We imported the dataset, and performed several procedures evaluating the employed amount of RAM, percentage of CPU, and time. For all tests we used an Intel Xeon E5-2440 (6 cores - 12 threads) with 32Gb of RAM.

Furthermore, we evaluated the effect that integration methods have on expression datasets to understand which technique yields better results. For this purpose, two analysis were carried out on (i) synthetic datasets and (ii) real datasets taken from The Cancer Genome Atlas (TCGA).

First, two synthetic datasets were generated to simulate Microarray and NGS experiments, respectively. The Microarray dataset was generated using the R package madsim [12], while the compcodeR [13] package was used for the NGS one. For both datasets, recommended parameters were used, generating 7 case samples and 7 controls. Both packages, in addition to expression matrices, provide the list of true Differentially Expressed Genes (DEGs) and their expected direction (upregulation or downregulation). Then, each dataset was randomly partitioned into two subset of samples and the integration methods were applied on them. Finally, DEGs were estimated using Limma, varying the p-value threshold from 0.001 to 0.05. Next, Receiver-Operating Characteristic (ROC) curves were built by comparing estimated DEGs with true ones. Curves were summarized by computing the Area Under the ROC Curve (AUC). Furthermore, the same test was performed on the original matrix without any integration procedure to obtain a baseline AUC, since some DEGs might not be detected due to Limma.

**Fig. 6:**
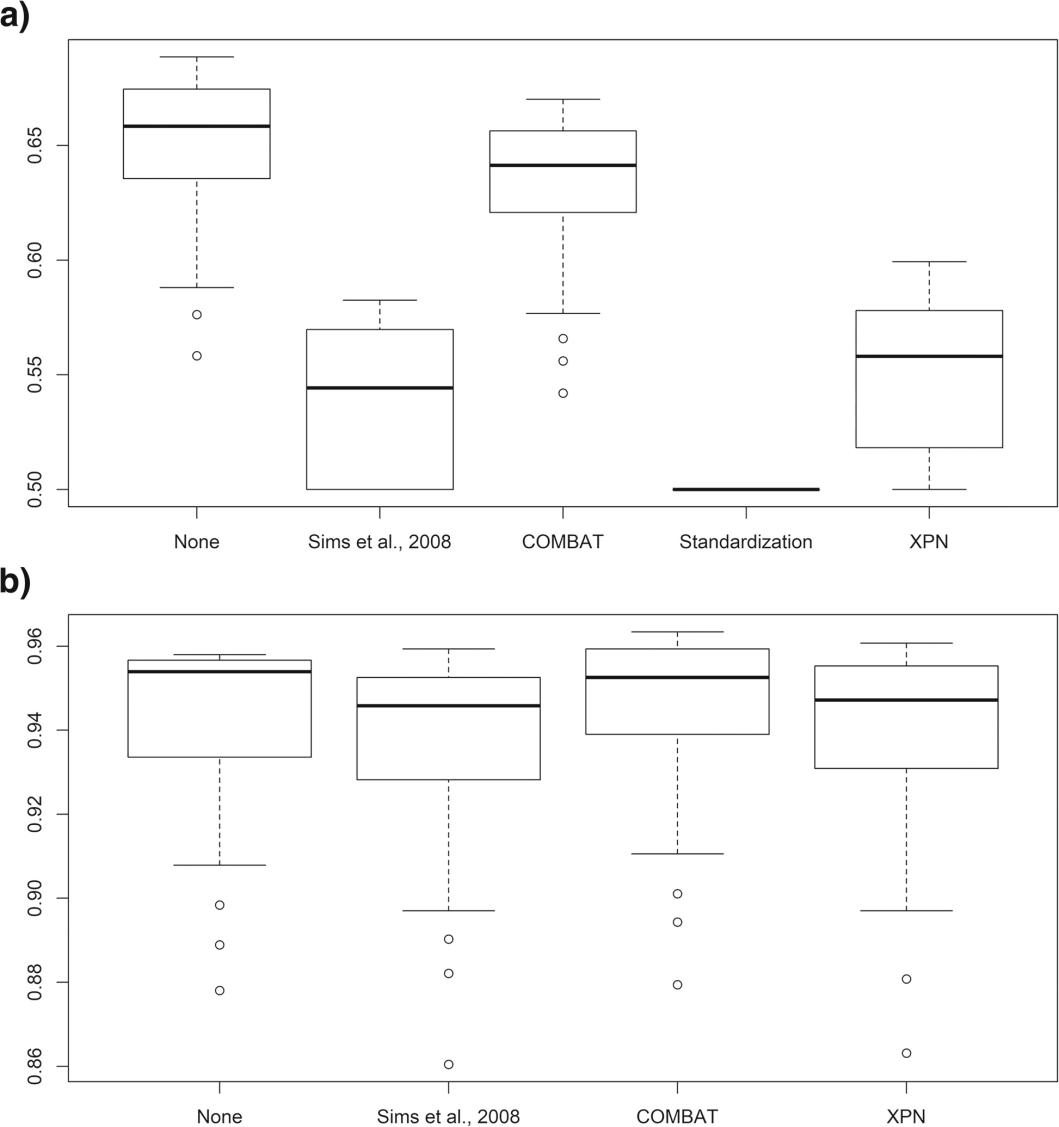
Effect of the integration procedures on synthetic expression datasets. The two datasets simulate Microarray data (**a**) and NGS data (**b**). Each synthetic dataset was randomly partitioned in two sets. Then, each integration procedure was applied and DEGs were computed varying the p-value threshold. Receiver-Operating Characteristic (ROC) curves were computed using true DEGs, and the performances were assessed by means of Area Under the ROC Curve (AUC)

For the simulated Microarray dataset (Fig. 6b), all methods obtain an AUC comparable to the baseline except for gene standardization which obtained ∼ 0.5. Therefore, we excluded the latter from Fig. 6b to allow a better visualization. For the simulated NGS dataset (Fig. 6a), the baseline method obtained a mean AUC greater than 0.65. Only COMBAT obtained a comparable value. The other three methods had significantly lower values.

**Fig. 7:**
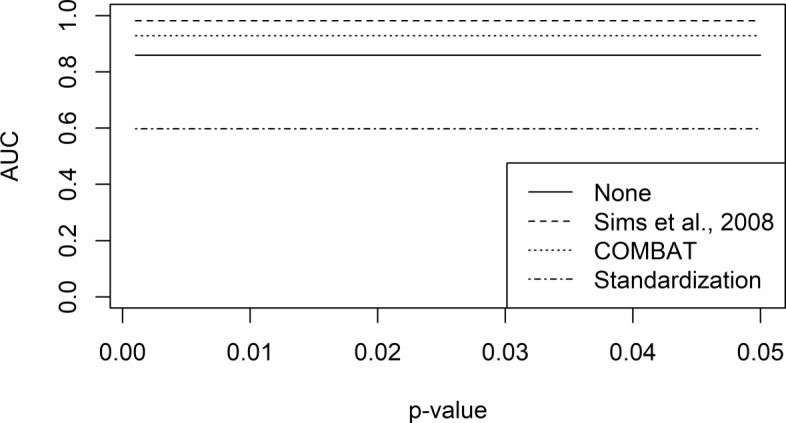
Effect of the integration procedures on TCGA LUSC datasets. Two datasets comprising of Illumina HiSeq-2000 and Affymetrix HG-U133A data were employed to evaluate the effect of the integration on two different platforms. After applying the integration procedure, DEGs were computed varying the p-value threshold. Receiver-Operating Characteristic (ROC) curves were computed using known DEGs, and the performances were assessed by means of Area Under the ROC Curve (AUC)

The second test was performed on the Lung Squamous Cell Carcinoma (LUSC) dataset obtained from TCGA. The dataset comprises of 552 RNA-seq samples (501 cases and 51 controls) obtained using the Illumina HiSeq-2000 sequencing platform. Furthermore, expressions measured using Affymetrix HG-U133A were available for 131 of the 501 cases. First we built a reference set by determining DEGs on all RNA-seq samples using Limma (*p*<0.01). Next, we removed the RNA-seq samples corresponding to the 131 microarray cases. This yielded two different dataset. Then, the two series of samples were processed using integration algorithms. Finally, the performances were evaluated using the above described methodology. Results show that both Sims et al., 2008 and COMBAT are capable to obtain results more accurate than the baseline approach (Fig. 7).

## Discussion

*TACITuS* is a user-friendly system providing a web interface designed to simplify the process of collection, pre-processing, selection, and integration of large transcriptomics datasets. The system also enables uploading of data to Galaxy Servers to allow further analysis.

**Fig. 8:**
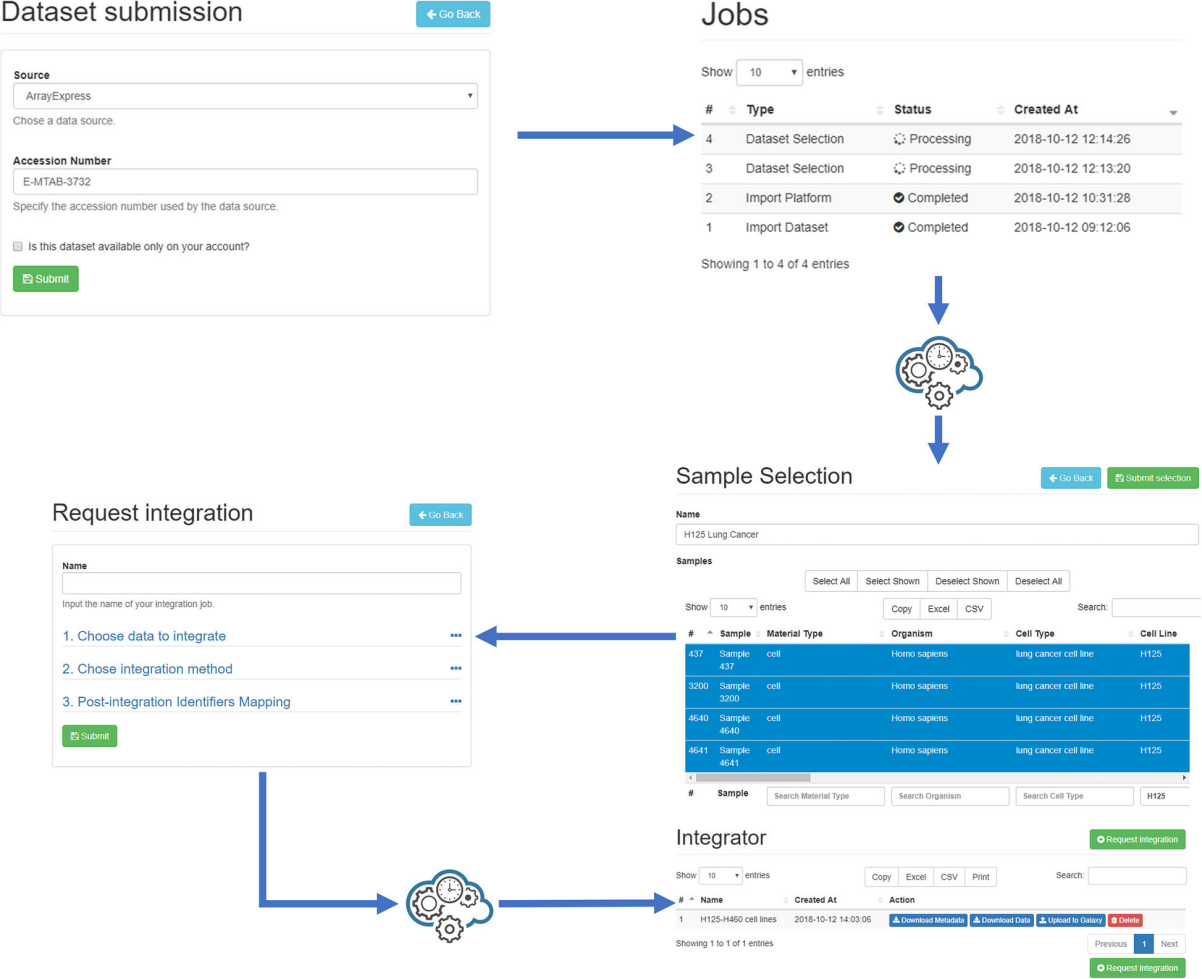
A common workflow in *TACITuS*. First, the user selects a dataset from a public database. As soon as the dataset is imported, selections on the samples can be performed. Finally, selection from multiple datasets can be integrated to obtain a single set of samples and their metadata

To show how our approach can easily manage large expression data, we perform a case study with the ArrayExpress dataset “A comprehensive human expression map” (E-MTAB-3732). The dataset has been imported through the “Submit Dataset” button in the “Datasets” panel. The system checked that all necessary parameters were specified. Next, the job was submitted to our analysis queue (see Fig. 8). In two hours, the system was able to import the dataset with minimal resource usage (~ 600*MB* of RAM and 8% sustained usage of a 6 cores CPU). At the end of the procedure, a notification was sent to the user, and the selection panel was enabled for the dataset (see Fig. 8).

**Fig. 9:**
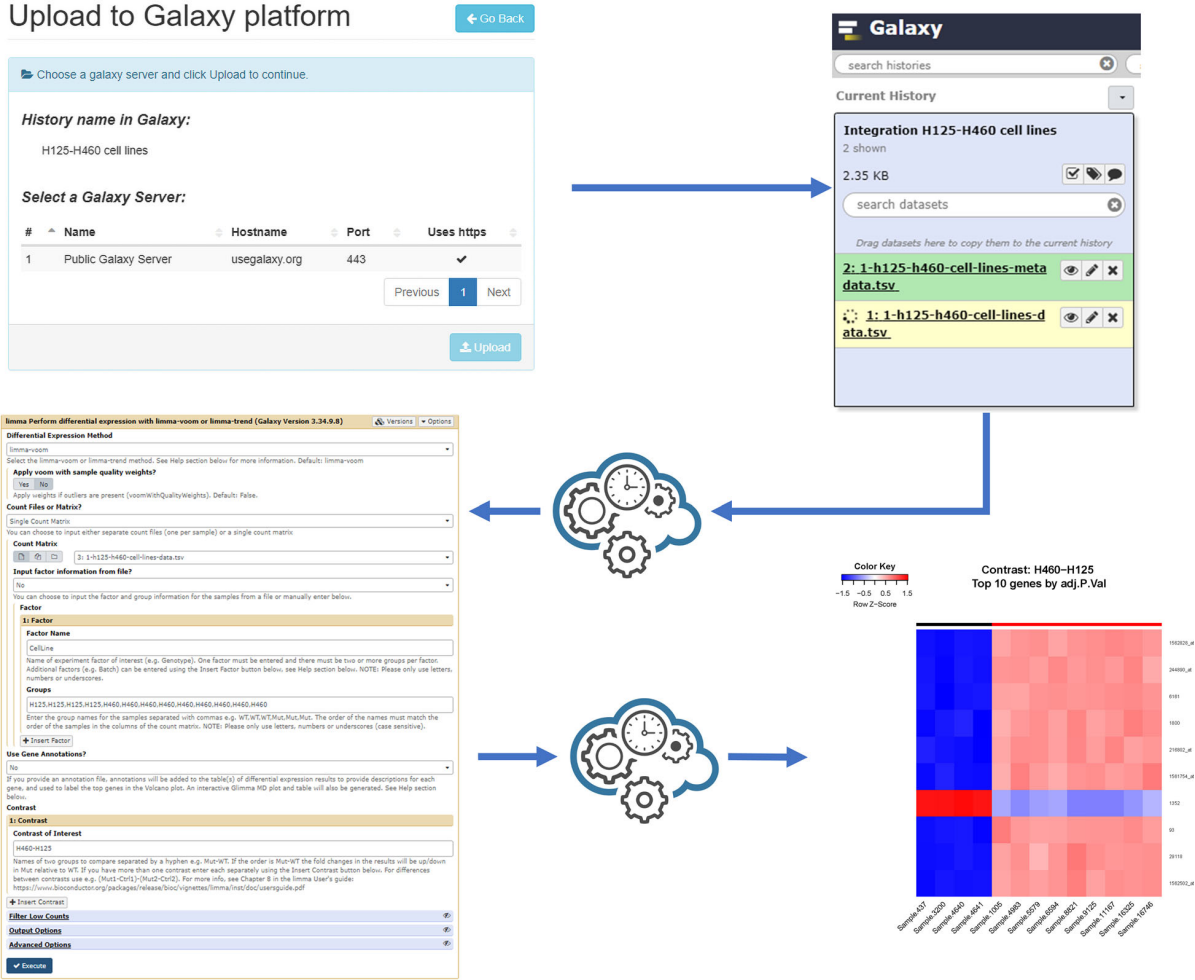
Galaxy Analysis Pipeline. A description of the galaxy analysis pipeline used for our case study. First data were uploaded in Galaxy using *TACITuS*. Next, we applied Limma to determine if any differentially expressed genes were present

We decided to select samples from two cell lines: H125 (lung cancer samples) and H460 (non-small cell lung cancer samples). These have been separately submitted to *TACITuS*, to obtain two different sub-datasets. The system was able to generate such subsets in one hour consuming only 370*MB* of RAM. Once the data become available, we used the integration tool to build a single dataset and map probe identifiers to Entrez Gene IDs (see Fig. 8). The procedure took only a few minutes using a modest amount of memory, approximately 1*GB* of RAM. Finally, we uploaded the data in the Galaxy Public Server, and performed a differential expression analysis using Limma (see Fig. 9).

Our tests show that *TACITuS* can handle large datasets with modest computational resources. Moreover, the simplified interface allows inexperienced users to manage large datasets. To evaluate integration algorithms impact, we performed several experiments on synthetic and real datasets. Experiments carried out on synthetic datasets showed how COMBAT is the only integration method capable to yield results comparable both on Microarray and NGS data. However, the chosen technique seems to have less impact in NGS rather than Microarrays. These results are also confirmed in the TCGA LUSC dataset. Both COMBAT and Sims et al., 2008 were able to obtain superior results compared to the baseline approach.

## Conclusions

*TACITuS* is a web app allowing users to manage and integrate data coming from ArrayExpress and NCBI GEO. Large transcriptomics datasets can be easily pre-processed, selected, and integrated. This enables many users to access large genome data usually available only to advanced bioinformaticians with large computational resources. The system allows also to upload data to the Galaxy platform for further analysis.

## Availability and requirements

**Project Name:***TACITuS*

**Project Home page:** https://tacitus.app/

**Bugs Reporting:** https://github.com/alaimos/tacitus/issues

**Operating system:** Platform independent

**Programming languages:** PHP, R, C++

**Other requirements:** All modern web browser

**License:** GNU GPL 3

**Other restrictions:** For non-academics, licence is needed

## Data Availability

The datasets analysed during the current study are available in the ArrayExpress repository (E-MTAB-3732), https://www.ebi.ac.uk/arrayexpress/experiments/E-MTAB-3732/.

## Abbreviations

ADF: Array design format
API: Application programming interface
AUC: Area under the ROC curve
DEG: Differentially-expressed gene
GEO: Gene expression omnibus
GPL: GNU general public license
IDF: Investigation description format
LUSC: Lung squamous cell carcinoma
MAGE-TAB: MicroArray gene expression tabular
MIAME: Minimum information about a microarray experiment
MINSEQE: Minimum information about a high-throughput nucleotide SEQuencing experiment
NCBI: National center for biotechnology information
NGS: Next-generation sequencing
ROC: Receiver-operating characteristic curve
SDRF: Sample and data relationship format
TACITuS: Transcriptomic data collector, integrator, and selector
TCGA: The cancer genome atlas
XPN: Cross-platform normalization
